# Intestinal Duplication and Hirschsprung's Disease: An Extremely Rare and Misleading Combination

**DOI:** 10.1055/s-0038-1675378

**Published:** 2018-12-26

**Authors:** Fabrizio Vatta, Alessandro Raffaele, Noemi Pasqua, Marco Brunero, Gloria Pelizzo, Luigi Avolio

**Affiliations:** 1 Department of Pediatric Surgery, Fondazione IRCCS Policlinico San Matteo, Pavia, Lombardia, Italy; 2 Department of Pediatric Surgery, Ospedale dei Bambini G Di Cristina, Palermo, Italy

**Keywords:** intestinal duplication, Hirschsprung's disease, intestinal occlusion

## Abstract

Hirschsprung's disease and, more rarely, intestinal duplication can both cause intestinal obstruction in neonates. The simultaneous occurrence of these two diseases is reported in only two studies, and in both cases, intestinal duplication was an incidental finding, as it had not determined clinical intestinal occlusion. This paper reports a unique case of coexistence of the two conditions, with both causing intestinal obstruction, delayed appropriate, and definitive surgical treatment.

## Introduction

Hirschsprung's disease (HD) is defined by the absence of enteric neurons in the distal part of the bowel. Neonates with HD usually present with a distended abdomen, feeding intolerance with bilious vomiting, and delayed passage of meconium. Failure to promptly diagnose HD can result in death secondary to recurrent enterocolitis. 1 2 Although its etiology is still unknown, several gene defects have been discovered. 3

Intestinal duplication (ID) is a rare congenital condition usually involving the small intestine, located on the mesenteric side of the bowel, with which it may share blood supply. Although it is symptomatic at birth in only 30% of cases, diagnosis is made before 2 years of life in up to 70% of patients. Symptoms vary as ID can affect any part of the gastrointestinal tract. 4 Many hypotheses have been made about its etiology, such as intrauterine hypoxia, partial twinning, split notochord syndrome, and remnants of embryological diverticula. 5 6 7

To date, no cases of intestinal obstructions simultaneously caused by both HD and ID have been reported. 8 9 Our study presents a unique case, where appropriate diagnosis and treatment were delayed due to the misleading presence of both ID and HD.

## Case Report

**Fig. 1:**
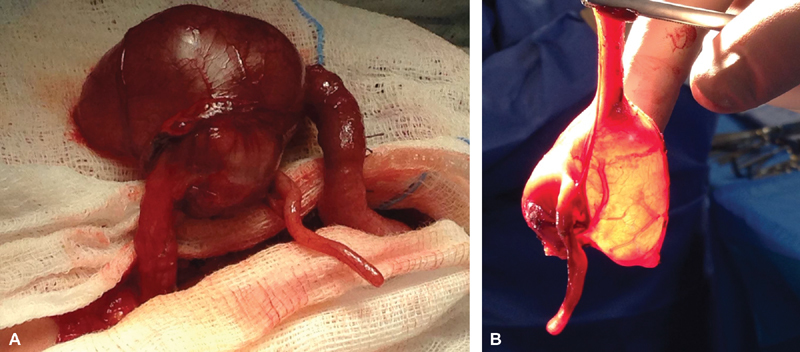
( **A** ) Occlusive duplication at the ileocecal junction. **(B)** Resected ileocecal region including intestinal duplication.

A full-term male neonate born at our Institute by induced vaginal delivery presented with bilious vomiting and abdominal distension starting from day 2 of life. X-ray revealed substantial distension of the small bowel, and abdominal ultrasound showed a simple cystic mass in the right hypochondrium (38 × 27 mm). Explorative laparotomy found an occlusive duplication at the ileocecal junction ( Fig. 1A ). Since resection of the duplication was not possible, we performed exeresis of the ileocecal region and end-to-end anastomosis ( Fig. 1B ). Gross pathology reaffirmed the diagnosis of ID and confirmed obstruction of the intestinal lumen.

Postsurgical course was characterized by recurrent bilious vomiting, abdominal distension, and constipation. At 1 month of life, due to exacerbation of his symptoms, the child underwent explorative relaparotomy: anastomotic stenosis and fibrous bands were excluded as the cause of occlusion. Instead, we found massive intestinal adherences, not apparently due to the previous surgery, as they were generalized and not localized, and were not causing bowel obstruction.

Despite a second surgical procedure, abdominal distension persisted, associated with the absence of spontaneous evacuation and impaired weight gain. Since postsurgical complications had already been excluded at the second laparotomy, we started suspecting HD. Another explorative laparotomy was planned a month later: ileostomy was performed along with multiple intestinal biopsies, which confirmed the diagnosis of short-segment HD (length of aganglionic bowel of ∼10 cm from the anal verge).

At nine months of life, the child successfully underwent transanal endorectal pull-through (De La Torre). Intestinal recanalization followed 3 months later. At 1 year follow-up, the child had normal bowel function, and no more episodes of abdominal distension have occurred.

## Discussion

Only two cases of co-occurring HD and ID are reported in the literature in English. The first, published in 2012, is that of a 35-year-old woman with a medical history of HD treated with Soave procedure at 29 months of life presenting with an adenocarcinoma originating from a rectal duplication. 8 The second, published in 2017, is that of a full-term female newborn in whom an ileal duplication was accidentally found when she underwent exploratory laparotomy to perform multiple full-thickness biopsies for HD. 9 We also found one case of association of cystic bowel duplication and intestinal neuronal dysplasia described in 1983. 10 Our case is the first in which co-occurring ID and HD have constituted a clinical challenge rather than an incidental finding since they both caused clinical bowel obstruction. The presence of one masked the presence of the other, delaying diagnosis and immediate appropriate therapy. In hindsight, the massive presence of adhesions encountered during the second laparotomy could have prompted suspicion of HD, as adhesions form secondary to Hirschsprung-associated enterocolitis. As far as etiopathogenesis is concerned, both pathologies have unclear causal factors, and many hypotheses have been proposed. One advanced for both conditions is intrauterine hypoxia, postulated in the pathogenesis of ID, 5 6 7 8 9 10 11 but also suggested as an interfering factor in ganglion cell migration in HD. 10 Although it is highly possible that the association of HD and ID is coincidental, it might be speculated that the coexistence of HD and ID is secondary to ischemic damage that occurs during embryogenesis and that interferes with both intestinal canalization and neural crest migration.

## Conclusion

The coexistence of ID and HD is extremely rare, and only two cases have been described in the English literature. As shown in our report, the presence of HD and ID together can mislead the diagnostic work-up and delay appropriate therapy.

## Notes

New Insights and the Importance for a Pediatric Surgeon When treating neonates, pediatric surgeons should always be alert to the possibility of unusual combinations of pathologies with a similar clinical presentation, where one condition may mask the other.
